# Visual influences on auditory spatial learning

**DOI:** 10.1098/rstb.2008.0230

**Published:** 2008-11-04

**Authors:** Andrew J. King

**Affiliations:** Department of Physiology, Anatomy and Genetics, University of OxfordSherrington Building, Parks Road, Oxford OX1 3PT, UK

**Keywords:** sound localization, spatial hearing, multisensory integration, auditory plasticity, behavioural training, vision

## Abstract

The visual and auditory systems frequently work together to facilitate the identification and localization of objects and events in the external world. Experience plays a critical role in establishing and maintaining congruent visual–auditory associations, so that the different sensory cues associated with targets that can be both seen and heard are synthesized appropriately. For stimulus location, visual information is normally more accurate and reliable and provides a reference for calibrating the perception of auditory space. During development, vision plays a key role in aligning neural representations of space in the brain, as revealed by the dramatic changes produced in auditory responses when visual inputs are altered, and is used throughout life to resolve short-term spatial conflicts between these modalities. However, accurate, and even supra-normal, auditory localization abilities can be achieved in the absence of vision, and the capacity of the mature brain to relearn to localize sound in the presence of substantially altered auditory spatial cues does not require visuomotor feedback. Thus, while vision is normally used to coordinate information across the senses, the neural circuits responsible for spatial hearing can be recalibrated in a vision-independent fashion. Nevertheless, early multisensory experience appears to be crucial for the emergence of an ability to match signals from different sensory modalities and therefore for the outcome of audiovisual-based rehabilitation of deaf patients in whom hearing has been restored by cochlear implantation.

## 1. Introduction

Our perception of the external world relies principally on vision and hearing. An ability to determine accurately and rapidly the location of a sound source is of great importance in the lives of many species. This is equally the case for animals seeking potential mates or prey as for those trying to avoid and escape from approaching predators. The process of localizing sounds also plays a key role in directing attention towards objects or events of interest, so that they can be registered by other senses, most commonly vision. Perhaps of most value in humans, spatial hearing can significantly improve the detection and, in turn, the discrimination of sounds of interest in noisy situations, such as a restaurant or bar. Consequently, preservation of this ability is of considerable importance in the rehabilitation of the hearing impaired. This review will consider the role of learning and plasticity in the development and maintenance of auditory localization and, in particular, the contribution of vision to this process.

## 2. Determining the direction of a sound source

In contrast to the visual and somatosensory systems, where stimulus location is encoded by the distribution of activity across the receptor surface in the retina or the skin, respectively, localizing a sound source is a highly complex, computational process that takes place within the brain. Because auditory space cannot be mapped onto the cochlea in the inner ear in the same way, the direction of a sound source has to be inferred from acoustical cues generated by the interaction of sound waves with the head and external ears (Blauert 1997; King *et al*. 2001). The separation of the ears on either side of the head is key to this, as sounds originating from a source located to one side of the head will arrive at each ear at slightly different times. Moreover, by shadowing the far ear from the sound source, the head produces a difference in amplitude level at the two ears. The level of the sound is also altered by the direction-specific filtering by the external ears, giving rise to spectral localization cues.

By themselves, each of these spatial cues is potentially ambiguous and is informative only for certain types of sound and regions of space. Thus, interaural time differences are used for localizing low-frequency sounds (less than approx. 1500 Hz in humans), whereas interaural level differences are more important at high frequencies. For narrow-band sounds, both binaural cues are spatially ambiguous, since the same cue value can arise from multiple directions known as ‘cones of confusion’ (Blauert 1997; King *et al*. 2001). Similarly, spectral cues are ineffective unless the sound has a broad frequency content (Butler 1986), in which case they enable the front–back confusions in the binaural cues to be resolved. Spectral cues also provide the basis for localization in the vertical plane and even allow some capacity to localize sounds in azimuth using one ear alone (Slattery & Middlebrooks 1994; Van Wanrooij & Van Opstal 2004). However, because this involves detection of the peaks and notches imposed on the sound spectrum by the external ear, the sound must have a sufficiently high amplitude for these features to be discerned (Su & Recanzone 2001). Moreover, under monaural conditions, these cues no longer provide reliable spatial information if there is uncertainty in the stimulus spectrum (Wightman & Kistler 1997). In everyday listening conditions, auditory localization cues are also likely to be distorted by echoes or other sounds. Accurate localization can therefore be achieved and maintained only if the information provided by the different cues is combined appropriately within the brain.

**Figure 1:**
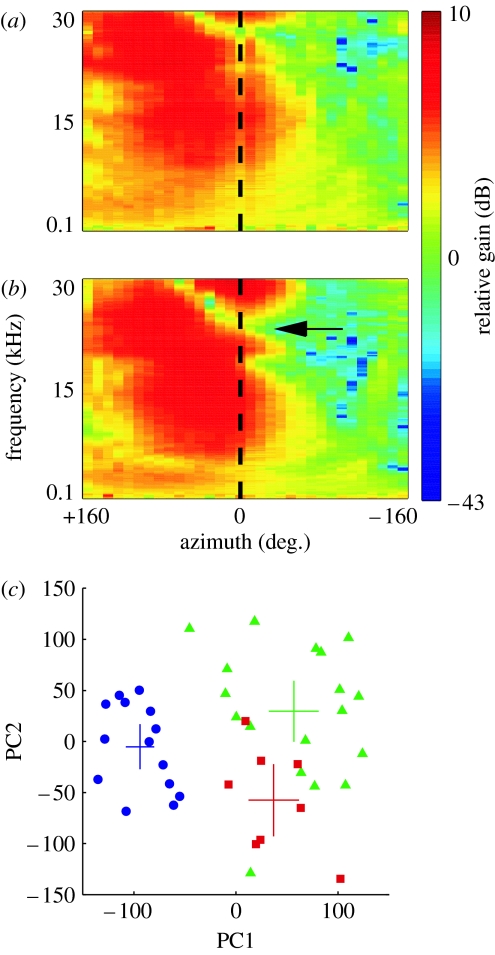
The acoustical cues that provide the basis for auditory localization change in value as the head and ears grow. Directional transfer functions (DTFs) from (*a*) an infant (post-natal day (P) 33) and (*b*) an adult ferret (*Mustela putorius*). Each plot shows how the gain in decibels of the external ear varies as a function of sound azimuth (*x*) and frequency (*y*) at a constant elevation (the animal's horizon). The difference in gain on either side of the midline (0°) is due to the acoustic shadowing effect of the head. The infant ear is less directional and features present in the adult, such as the high-frequency notch (arrowed in (*b*)), are shifted to higher frequencies. (*c*) Age-related differences are quantified for a large number of animals by converting each DTF to a vector and performing a principal components analysis on the population of DTF vectors. Data are plotted along the first two principal components (which cumulatively explain 56% of the variance). Points are labelled according to the age of the animal and each animal is represented by two data points, one for each ear. The large crosses indicate the mean and 95% confidence intervals for each distribution. There is a clear distinction between the infant (blue, P33–P37) and adult points (green) with no overlap between the distributions. Juvenile animals (red, aged approx. P50 (P49–P51)) have intermediate DTFs that overlap with the adult distribution, indicating that spectral cues approach maturity three to four weeks after the onset of hearing in this species. Adapted from Campbell *et al*. (2008).

The values of the localization cues vary not only with the properties of the sound, but also with the dimensions of the head and external ears. Thus, during development, the cue values associated with any given direction in space will gradually change while these structures are growing (Moore & Irvine 1979; Clifton *et al*. 1988; Schnupp *et al*. 2003; Campbell *et al*. 2008; figure 1). Consequently, representations of sound-source location in the brain cannot become fully mature until the auditory periphery has stopped growing. Moreover, the adult values attained once development is complete will vary from one individual to another according to differences in the size, shape and separation of the ears. This implies that listeners must learn by experience to localize with their own ears, a view that is supported by the finding that humans can localize headphone signals that simulate real sound sources more accurately when these are derived from measurements made from their own ears than from the ears of other individuals (Wenzel *et al*. 1993; Middlebrooks 1999).

Accurate auditory localization relies on non-acoustic factors too. Because the coordinates of auditory space are centred on the head and ears, information must be provided by the vestibular and proprioceptive senses about the orientation and motion of these structures (Goossens & Van Opstal 1999; Vliegen *et al*. 2004). Moreover, a congruent representation of the external world has to be provided by the different senses, so that the objects registered by more than one modality can be reliably localized and identified. In the case of vision and hearing, this means that activation of a specific region of the retina corresponds to a particular combination of monaural and binaural localization cues values. Because most animals can move their eyes, that relationship is not fixed. Consequently, the neural processing and perception of auditory spatial information is also influenced by the direction of gaze (Zwiers *et al*. 2004; Bulkin & Groh 2006; Razavi *et al*. 2007).

## 3. Visual influences on the accuracy of auditory localization

Although sound sources can obviously be localized on the basis of auditory cues alone, localization accuracy improves if the target is also visible to the subject (Shelton & Searle 1980; Stein *et al*. 1989). This is an example of a more general phenomenon by which the central nervous system can combine inputs across the senses to enhance the detection, localization and discrimination of stimuli and speed up reactions to them. Cross-modal interactions also occur when conflicting information is provided by different senses. For instance, it is frequently the case when listening to someone's voice that we also see their lips moving, which, particularly in noisy situations, can improve speech intelligibility (Sumby & Pollack 1954). But when the visual and auditory signals no longer match, as occurs when viewing someone articulating one speech syllable while listening to another, listeners typically report hearing a third syllable that represents a combination of what was seen and heard (the ‘McGurk effect’; McGurk & MacDonald 1976).

Conflicting visual cues can also influence the perceived location of a sound source. Thus, the presentation of synchronous but spatially disparate visual and auditory targets tends to result in mislocalization of the auditory stimulus, which is perceived to originate from near the location of the visual stimulus (Bertelson & Radeau 1981). Visual capture of sound-source location forms the basis for the ventriloquist's illusion and explains why we readily associate sounds with their corresponding visual events on a television or cinema screen, rather than with the loudspeakers located to one side. This is usually thought to arise because, in contrast to the ambiguous and relatively imprecise cues that underlie auditory localization, the retina provides the brain with high-resolution and reliable information about the visual world. However, if visual stimuli are blurred so that they become harder to localize, the illusion can work in reverse, with sound capturing vision (Alais & Burr 2004). Along with other studies (Ernst & Banks 2002; Battaglia *et al*. 2003; Heron *et al*. 2004), this finding suggests that rather than vision having an inherent advantage in spatial processing, the weighting afforded to different sensory cues when they are integrated by the brain varies according to how reliable they are.

**Figure 2:**
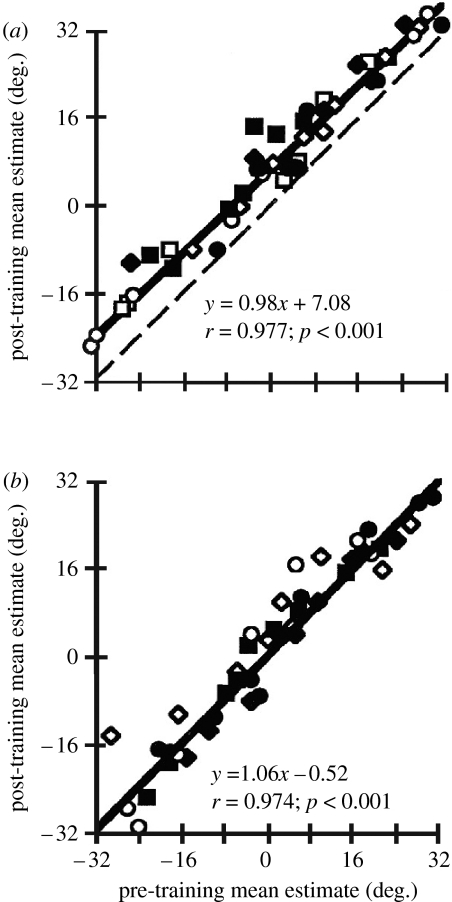
The ventriloquism after-effect. Regression of mean auditory localization judgments taken before (pre-training, *x*-axis) and after (post-training, *y*-axis) exposing human subjects to spatially misaligned auditory and visual stimuli. (*a*) Data obtained after conditioning with an 8° mismatch between the stimuli. The dashed line indicates perfect correlation between the pre- and post-training estimates. In this case, the auditory localization estimates have been shifted in the direction of the previously present visual stimulus, so the regression line lies above the dashed line. (*b*) Data obtained after conditioning with a 0° mismatch between the stimuli. There is no difference between pre- and post-training estimates and the dashed line is hidden by the thicker solid regression line. Adapted and reproduced with permission from Recanzone (1998). Copyright (1998) National Academy of Sciences, USA. Open and filled squares, subject 1; open and filled diamonds, subject 2; open and filled circles, subject 3 (open symbols, 750 Hz; filled symbols, 3 kHz).

Nevertheless, visual localization is normally more accurate than sound localization and therefore tends to dominate conflicts between the two modalities, thereby enabling, at least within certain limits, spatially misaligned cues to be perceived as if they originate from the same object or event. Repeated presentation of consistently misaligned cues results in a shift in the perception of auditory space that can last for tens of minutes once the visual stimulus is removed (figure 2). This is known as the ventriloquism after-effect and has been observed in both human (Radeau & Bertelson 1974; Recanzone 1998; Lewald 2002) and non-human (Woods & Recanzone 2004) primates. Short-term changes in auditory localization can also be induced in humans by compressing the central part of the visual field for 3 days with 0.5× lenses (Zwiers *et al*. 2003). These studies highlight the dynamic nature of auditory spatial processing in the mature brain, which allows the perceived location of sound sources to be modified in order to conform to changes in visual space.

## 4. Visual–auditory interactions in the brain

Revealing where and how multisensory information is combined and integrated in the brain is critical to understanding the basis by which visual inputs influence auditory perception and behaviour. Until recently, multisensory convergence was thought to be the preserve of specific cortical and subcortical brain regions. It is now clear, however, that many cortical regions receive afferent inputs from more than one of the senses, including primary areas that were previously thought to be modality specific. This seems to be particularly the case in the auditory cortex, where sensitivity to visual stimulation has been demonstrated in humans (Calvert *et al*. 1999; Giard & Peronnet 1999; Molholm *et al*. 2002), non-human primates (Schroeder & Foxe 2002; Brosch *et al*. 2005; Ghazanfar *et al*. 2005; Kayser *et al*. 2007), ferrets (Bizley *et al*. 2007) and rats (Wallace *et al*. 2004*a*).

One likely function for these multisensory interactions has been revealed by Ghazanfar *et al*. (2005) who found that responses to vocalizations in monkey auditory cortex could be either enhanced or suppressed when the animals viewed the corresponding facial expressions, whereas this was less likely to be the case if the image of the face was replaced by a disc that was flashed on and off to mimic the mouth movements. It has been suggested that visual and somatosensory inputs can modulate the phase of oscillatory activity in the auditory cortex, potentially amplifying the response to related auditory signals (Schroeder *et al*. 2008). A facilitatory role for these inputs in sound localization has also been proposed (Schroeder & Foxe 2005), a possibility supported by the finding that visual inputs can increase the amount of spatial information conveyed by neurons in auditory cortex (Bizley & King 2008). As the cortex is necessary for normal sound localization (Recanzone & Sutter 2008), it seems likely that such interactions could underlie the visual capture of auditory space perception. Indeed, the observation that the ventriloquism after-effect does not seem to transfer across sound frequency (Recanzone 1998; Lewald 2002; Woods & Recanzone 2004, but see also Frissen *et al*. 2005 for a different result) implies that early, tonotopically organized regions are involved.

## 5. Visual guidance of auditory spatial processing during development

In addition to recalibrating auditory space whenever temporary spatial mismatches occur, vision plays an important role in guiding the maturation of the auditory spatial response properties of neurons in certain regions of the brain. This has been demonstrated most clearly in the superior colliculus (SC) in the midbrain, where visual, auditory and tactile inputs are organized into topographically aligned spatial maps (King 2004). This arrangement allows each of the sensory inputs associated with a particular event to be transformed into appropriate motor signals that control the direction of gaze. Where individual SC neurons receive converging multisensory inputs, the strongest responses can often be generated to combinations of stimuli that occur in close temporal and spatial proximity, which, in turn, appears to improve the accuracy of orienting responses (Stein & Stanford 2008). As with the perception of space, maintenance of intersensory map alignment in the SC requires the incorporation of eye-position information in order to allow for differences in the reference frames used to specify visual and auditory spatial signals (Jay & Sparks 1987; Hartline *et al*. 1995; Populin *et al*. 2004).

**Figure 3:**
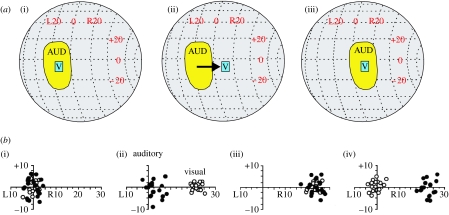
Visual experience shapes the developing auditory localization pathway in the barn owl (*Tyto alba*). (*a*) Effects of prism rearing on the auditory spatial receptive fields of neurons in the optic tectum. (i) Visual (V) and auditory (AUD) receptive fields are normally in close correspondence. (ii) Placing prisms that displace the visual field to the right by 23° disrupts the alignment of these receptive fields. (iii) After young owls have worn the prisms for eight weeks, the auditory receptive field has shifted so that it becomes realigned with the visual receptive field. Adapted from Knudsen & Brainard (1991). (*b*) Adjustment of auditory orienting responses in a prism-reared owl. Head-orienting responses to visual (open circles) or auditory (filled circles) targets plotted with respect to the location of the stimulus. Owls normally make accurate head turns towards either stimulus. Prisms immediately shift the visual responses, but have no effect on the auditory responses. However, after 42 days of experience with the prisms, the auditory responses have shifted to match the optical displacement of the visual field, presumably as a consequence of the changes that take place in the optic tectum. When the prisms are removed, normal visual responses are restored, although it takes several weeks for the auditory orienting responses to recover ((i) before prisms, (ii) after 1 day with prisms, (iii) after 42 days with prisms and (iv) prisms removed). Adapted from Knudsen & Knudsen (1990).

The dominant role played by vision in aligning the sensory maps in the SC has been demonstrated by altering the spatial relationship between auditory localization cues and directions in visual space. This has been achieved by surgically inducing a persistent change in eye position (King *et al*. 1988), by the use of prisms that laterally displace the visual field representation (Knudsen & Brainard 1991; figure 3), and, most recently, by maintaining young animals in the dark and periodically exposing them to temporally coincident but spatially incongruent visual and auditory stimuli (Wallace & Stein 2007). In each case, a corresponding shift in the neural representation of auditory space is produced, which, at least in prism-reared owls, involves a rewiring of connections within the midbrain (DeBello *et al*. 2001). In addition to these long-lasting effects on auditory spatial tuning, behavioural studies in owls have shown that prism experience induces equivalent changes in the accuracy of auditory head-orienting responses (Knudsen & Knudsen 1990; figure 3).

Studies in human infants have shown that certain multisensory abilities emerge at different stages within the first year of life (Neil *et al*. 2006; Kushnerenko *et al*. 2008). However, the capacity to integrate different sensory cues in a statistically optimal fashion emerges much later, at approximately 8 years of age (Gori *et al*. 2008; Nardini *et al*. 2008). Before this, one or other sense seems to dominate, which is potentially useful if one sense is used during development to calibrate another, as in the influence of vision on auditory spatial processing.

## 6. Auditory spatial learning in adulthood

Early studies in barn owls showed that altered vision results in adaptive auditory plasticity during a sensitive period of development, but not in older animals (Knudsen & Knudsen 1990; Knudsen & Brainard 1991). This is consistent with other studies, including those in mammals, which have explored the role of experience in the development and maintenance of sound localization mechanisms (King *et al*. 2000). Nevertheless, more recent experiments in owls have shown that the capacity for prism experience to induce long-term modifications of both the auditory space map (Brainard & Knudsen 1998; Knudsen 1998; Bergan *et al*. 2005) and localization behaviour (Brainard & Knudsen 1998) can be extended to older animals under certain conditions.

**Figure 4:**
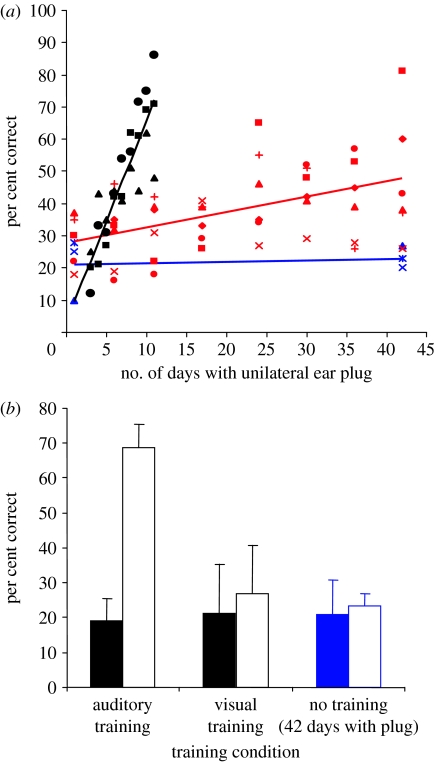
Auditory localization adaptation is accelerated by modality-specific training. (*a*) Change in performance (averaged across all speaker locations) over time in three groups of ferrets with unilateral earplugs. Each symbol represents a different animal. No change was found in trained ferrets that received an earplug for six weeks, but were tested only at the start and end of this period (*n*=3, blue symbols and regression line). Two groups of animals received an equivalent amount of auditory training while the left ear was occluded. Although the earplug was in place for less time, a much faster rate of improvement was observed in the animals that received daily training (*n*=3, black symbols and regression line) compared with those that were trained every 6 days (*n*=6, red symbols and regression line). (*b*) Mean (±s.d.) auditory localization scores at the start (filled bars) and end (open bars) of the plugging period for the animals that received daily auditory training for 10 days (black symbols in (*a*)), another group of auditory-trained ferrets that performed a visual localization task every day while the left ear was occluded for the same period (*n*=3), and the three ferrets that received no training during a six-week period of monaural occlusion (blue symbols in (*a*)). The only significant improvement in sound localization was found for the auditory-trained animals. Adapted from Kacelnik *et al*. (2006).

It is also now clear that the mature mammalian brain is capable of relearning to localize sound in the presence of substantially altered auditory spatial cues. This has been demonstrated in humans by placing moulds into the external ears in order to alter the spectral cues corresponding to different directions in space (Hofman *et al*. 1998; Van Wanrooij & Van Opstal 2005). As expected, this manipulation immediately impaired localization in the vertical plane. However, response accuracy subsequently recovered over the course of a few weeks. No after-effect was observed following removal of the moulds, indicating that this recalibration in the neural processing of spectral cues did not interfere with the capacity of the brain to use the cue values previously experienced by the subjects. In a similar vein, Kacelnik *et al*. (2006) showed that altering binaural cues by reversible occlusion of one ear greatly reduces the accuracy with which adult ferrets can localize sounds in the horizontal plane. Again, performance rapidly improved over the next week or so, but only if the animals received auditory localization training after the earplug had been introduced. Indeed, the extent and rate of improvement were determined by the frequency of training (figure 4). Compared with the initial pattern of errors induced by the earplug, a very small and transient after-effect was seen following its removal. This indicates that plasticity in this task is unlikely to be attributed to the animals learning a new association between the altered binaural cues and directions in space. Instead, it appears that the brain is capable of reweighting the different auditory cues according to how reliable or consistent they are in a manner that resembles the optimal integration of multisensory cues in ventriloquism.

Given the evidence that vision can recalibrate auditory localization both during infancy and in later life, it seems reasonable to assume that visual cues provide a possible source of sensory feedback about the accuracy of acoustically guided behaviour, which might therefore guide the plasticity observed when localization cues are altered. Indeed, if they are also deprived of vision, smaller adaptive changes occur in the tuning of midbrain neurons in owls raised with one ear occluded (Knudsen & Mogdans 1992). Moreover, normally sighted birds that have adapted to monaural occlusion fail to recover normal localization behaviour following earplug removal if vision is prevented at the same time (Knudsen & Knudsen 1985).

**Figure 5:**
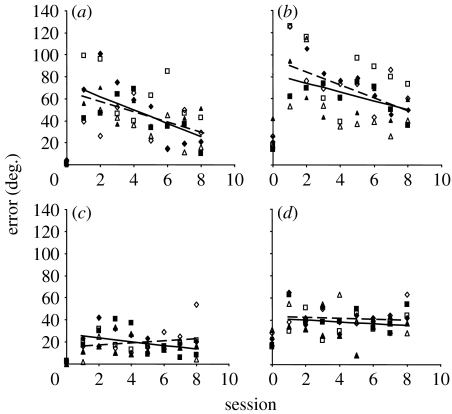
Relearning to localize sound in the presence of a unilateral conductive hearing loss does not require visual feedback. Data from three normally sighted (open symbols) and three visually deprived (filled symbols) ferrets showing the magnitude of the unsigned errors averaged across speaker location in the left ((*a*,*b*) side of plugged ear) and right ((*c*,*d*) side of open (unplugged) ear) hemifields. Each symbol represents a different animal. Data are shown for the session prior to the insertion of the earplug (session 0) and for eight sessions, carried out at 6-day intervals, with the left ear occluded (sessions 1–8). (*a*,*c*) 1000 ms, (*b*,*d*) 40 ms. Adapted from Kacelnik *et al*. (2006).

However, visual feedback is neither sufficient nor required for recalibrating auditory space in response to altered binaural cues in adult mammals. Kacelnik *et al*. (2006) found that ferrets with unilateral earplugs do not recover auditory localization accuracy if they are trained on a visual, rather than an auditory, localization task (figure 4*b*), highlighting the requirement for modality-specific training, while normal plasticity was seen in response to auditory training in animals that had been visually deprived from infancy (figure 5). It has been suggested that these results can be accounted for by unsupervised sensorimotor learning, in which the dynamic acoustic inputs associated with an animal's own movements help build up a stable representation of auditory space (Aytekin *et al*. 2008). Although vision is not essential for the relearning of accurate sound localization by monaurally occluded ferrets, it is certainly possible that training with congruent multisensory cues might result in faster learning than that with auditory cues alone, as recently demonstrated in humans for a motion detection task (Kim *et al*. 2008).

## 7. Visual–auditory interactions following sensory deprivation: implications for cochlear implantation

There is both behavioural and physiological evidence that early loss of vision can interfere with the maturation of certain aspects of auditory spatial processing (Withington-Wray *et al*. 1990; King & Carlile 1993; Zwiers *et al*. 2001; Wallace *et al*. 2004*b*). Nevertheless, there is no doubt that accurate sound localization can develop in the absence of vision (figure 5) and, in some cases, even surpass the performance of individuals with normal sight (King & Parsons 1999; Röder *et al*. 1999; Gougoux *et al*. 2005; Lewald 2007). Enhanced auditory capacities could result from changes within the auditory pathway (Korte & Rauschecker 1993; Elbert *et al*. 2002), perhaps reflecting the greater attention paid to the auditory modality. However, the recruitment of visual cortex also seems to be involved in the superior auditory localization performance of blind individuals (Weeks *et al*. 2000; Gougoux *et al*. 2005), which is presumably made possible either by establishing novel functional connections from auditory to visual brain areas or by unmasking connections that are now known to exist normally.

Although the emergence of heightened abilities in the use of the remaining senses in the blind or deaf is clearly advantageous, this has important implications for the capacity of the brain to process inputs from the missing sense, should these be restored, and to coordinate those inputs with the intact sensory modalities. Thus, while cross-modal reorganization of auditory cortex appears to facilitate the perception of visual speech in the deaf, it may limit the capacity of these individuals to make use of restored auditory inputs provided by cochlear implants (Doucet *et al*. 2006; Lee *et al*. 2007). Another important consideration is the effect of early sensory deprivation on the subsequent capacity to bind visual and auditory signals. Early loss of vision has been reported to impair the ability of the central nervous system to combine and integrate multisensory cues (Wallace *et al*. 2004*b*; Putzar *et al*. 2007). Synthesis of auditory and visual information can be achieved, however, if sensory function is restored early enough. Thus, congenitally deaf children fitted with cochlear implants within the first two and a half years of life exhibit the McGurk effect, whereas, after this age, auditory and visual speech cues can no longer be fused (Schorr *et al*. 2005). Interestingly, patients with cochlear implants received following postlingual deafness—who presumably benefitted from multisensory experience early in life—are better than listeners with normal hearing at fusing visual and auditory signals, thereby improving speech intelligibility in situations where both sets of cues are present (Rouger *et al*. 2007).

While cochlear implantation has enabled many profoundly deaf patients to recover substantial auditory function, including an ability to converse by telephone, they usually have difficulty in localizing sounds and perceiving speech in noisy conditions. This is because, in the vast majority of cases, only one ear is implanted. However, if hearing is restored bilaterally by implanting both ears, these functions can be recovered or, in the case of the congenitally deaf, established for the first time (Litovsky *et al*. 2006; Long *et al*. 2006). On the basis of the highly dynamic way in which auditory spatial information is processed in the brain, it seems certain that the capacity of patients to interpret the distorted signals provided by bilateral cochlear implants will be enhanced by experience and by training strategies that encourage their use in localization tasks. Moreover, provided that they can integrate multisensory inputs, training with congruent auditory and visual stimuli should be particularly useful for promoting adaptive plasticity in the auditory system of these individuals.
